# Rose Pearl

**Published:** 1869-01

**Authors:** 


### Editorial.

### ROSE PEARL.

We are having frequent inquiries in regard to this material as a base for artificial teeth. We will endeavor to answer some of them, so far as our limited experience will justify. We first saw this work more than two years ago, but have had personal experience with it only within the last few months, and our experience thus far is very gratifying, and coincides entirely with some others in our immediate vicinity, who have also, to a very considerable extent, been testing its merits. This style of work has some very desirable qualities. In regard to the durability of the material, we have strong faith that it will prove to be equal to any material now in use for this purpose; we do not mean by this that it is as unchangeable as gold or platinum in substance, but with these materials, as every one of experience knows, artificial dentures, though well constructed, are, in many cases, of but temporary duration, for teeth break from them very readily, and, indeed, we have oftentimes seen gold plates broken entirely through, and frequently none of the metals are wholly compatible with the living tissues upon which they are placed.

In the use of Rose Pearl as a base, the teeth are far more firmly attached than they can be upon gold plate; indeed, with these dentures, the breaking of a tooth is a very rare occurrence. The teeth, for the most part, are very firmly embraced by the material; and it possesses sufficient elasticity to accommodate any sudden jar. It is almost impossible to break the plate by bending or twisting. The plates may be made almost as thin as gold plates, and still have sufficient strength, so that there is an entire absence of the thickness and clumsiness attending rubber plates. It is lighter than any other style of work. The material itself constitutes the artificial gum, so that teeth without porcelain gums are used. This greatly facilitates the work of construction, and permits a far greater variety in the arrangement of teeth than it is possible to obtain with the ordinary blocks or sections used in rubber work.

Many questions are asked in regard to the method of working it. Experience warrants the assertion that it is about as easily worked, when the process is well understood, as any thing else upon which artificial teeth have been mounted.

One gentleman who has been using it but two months, says he now makes it easier than he ever did rubber work.

The method of construction doubtless will be much modified and improved; indeed, little points of improvement are being made almost every day, which will certainly ultimate in rendering the process a very simple and easy one.

Dr. McClelland, the inventor, is not putting this style of work into the hands of the profession as rapidly as we think he should; he desires to perfect and simplify as much as possible before putting it out. We are not certain that this is the best course; if fifty or a hundred good, careful experimenters were at work, the progress most probably would be far more rapid; at least it has proved so with other things.

We trust that this style of work may be taken up by the profession generally at the earliest possible period, and let whatever there is of good in it be brought out and made useful.

